# Macrophage Depletion Protects against Cigarette Smoke-Induced Inflammatory Response in the Mouse Colon and Lung

**DOI:** 10.3389/fphys.2018.00047

**Published:** 2018-02-12

**Authors:** Dahae Lim, Woogyeong Kim, Chanju Lee, Hyunsu Bae, Jinju Kim

**Affiliations:** ^1^Department of Korean Physiology, College of Pharmacy, Kyung Hee University, Seoul, South Korea; ^2^Department of Science in Korean Medicine, Kyung Hee University, Seoul, South Korea

**Keywords:** cigarette smoke, clodronate liposome, intestinal inflammation, macrophage, pulmonary inflammation

## Abstract

Cigarette smoke (CS) is considered as a major risk factor for pulmonary and intestinal inflammation. CS leads to macrophage infiltration in the mucosae of the lung and colon, inducing the uncontrolled secretion of inflammatory mediators, and thus promoting inflammatory response. In this study, we investigated whether macrophage depletion modulates cigarette smoke (CS)-induced inflammatory response in both the lung and colon. The mice were exposed to CS for 30 min, after which they were rested in a fresh air environment for 30 min. The total duration of exposure to CS was 2 h per day for 4 weeks. Macrophage depletion state was made with the injection of clodronate containing liposome. Individual body weights were measured twice a week, and the mice were sacrificed on day 28. Hematoxylin and eosin (H&E) staining was performed in the lung and colon tissue to determine histological changes. Inflammatory mediators' synthesis was analyzed using ELISA and western blotting. Clodronate liposome treatment ameliorated pathological changes associated with the infiltration of immune cells in the lung and colon. Also, clodronate liposome injected mice showed significantly lower level of inflammatory mediators, including cytokines and chemokine and proteases. Our results indicated that macrophage depletion by clodronate liposome treatment attenuates CS-induced inflammatory response in both the lung and colon.

## Introduction

Inflammation is a necessary defense mechanism against pathogen invasion from exterior environment. Various immune cells infiltrate into the inflamed area after pathogen invasion is detected in the body. Among these immune cells, macrophages play a central role in the inflammatory response. Macrophages accumulate in the inflamed tissue, removing infectious agents, resolving inflammation, and eliminating dead cells. They also repair damaged tissue during the inflammatory process by secreting multiple inflammatory mediators, including cytokines, chemokine and proteases. However, it is known that the hypersecretion of inflammatory mediators from the overactivated macrophages may result in inflammatory disorders (Koh and DiPietro, 2011; Jou et al., 2013; Dunster, 2016). Macrophages play a crucial role in controlling the mucosal immune balance (O'Donnell et al., 2006; Mann and Li, 2014; Indira et al., 2015). Therefore, macrophage dysfunction is an important factor in the pathogenesis of mucosal inflammatory diseases. Activated macrophages contribute to inflammatory progression through excessive secretion of inflammatory cytokines, chemokine, and proteases in the mucosal tissue such as the lung and colon (Heinsbroek and Gordon, 2009; Dunster, 2016; Belchamber and Donnelly, 2017). Many recent studies have reported therapeutic activities of macrophage depletion by clodronate liposome treatment in different pathological conditions. Golbar et al. reported that macrophage depletion attenuated alpha-naphthylisothiocyanate-induced biliary fibrosis (Golbar et al., 2017). Ren et al. reported that macrophage depletion diminished implant-wear-induced inflammatory osteolysis (Ren et al., 2008). Carlos et al. reported that macrophage depletion curtailed chronic cyclosporine A nephrotoxicity (Carlos et al., 2010).

Cigarette smoke (CS) is known to contain hundreds of toxic substances and carcinogens. CS can induce serious health problems, from which death may result in severe cases (Lee et al., 2012). In particular, CS is thought to be the leading cause of acute or chronic inflammation in the lung and colon, and is significantly associated with chronic obstructive pulmonary disease (COPD) and inflammatory bowel disease (IBD) (Martey et al., 2004; Mahid et al., 2006; Lunney et al., 2015). CS exposure elicits the influx of immune cells into the bronchi and the intestinal mucosa, and among all, macrophages secrete inflammatory cytokines, chemokines, and proteases in a disorderly manner (Kirkham et al., 2003; Heinsbroek and Gordon, 2009; Belchamber and Donnelly, 2017). There are numerous evidence supporting that CS exposure triggers macrophage recruitment and activation, thus responsible for inflammatory disorders (Thomas et al., 1978; Kirkham et al., 2003). Recently, a study by Pérez-Rial et al. (2013) established the importance of pro-inflammatory monocytes in lung tissue damaging after depleting alveolar macrophage and monocytes in blood. However, up to date, the effect of macrophage depletion in inflammatory response has not been fully elucidated. Therefore, our study investigated the effect of clodronate-liposome-induced macrophage depletion in reducing the pulmonary and intestinal inflammatory response using a cigarette smoking murine model.

## Materials and methods

### Animals

Female C57BL/6 mice (aged 7 weeks) were purchased from Orient Bio Inc. (Seongnam-si, Korea). Six mice were maintained together in one cage under pathogen-free conditions at 24°C with 55% humidity, and were provided a standard sterile rodent diet (Purina Mills, St. Louis, MO, USA) with water given *ad libitum*. The study was approved by the Kyung Hee University animal care and use committee, and all of the experiments were performed in accordance with the approved animal protocols and guidelines established by Kyung Hee University (KHUASP (SE)-12-015).

### Injection of clodronate liposome

Clodronate liposome and control liposome were purchased from FormuMax Scientific Inc. (Sunnyvale, CA, USA). 10 μl/kg of clodronate containing liposome (CL) or empty liposome (EL) was intraperitoneally injected 4 days before first CS exposure and 5 μl/kg of CL or EL was given by i.p. injection every 4 days during experiment for longer macrophage depletion. The injected doses and intervals of Clodronate liposome and control liposome were determined by following the protocol of FormuMax Scientific Inc. (Sunnyvale, CA, USA).

### Cigarette smoke exposure

**Figure 1:**
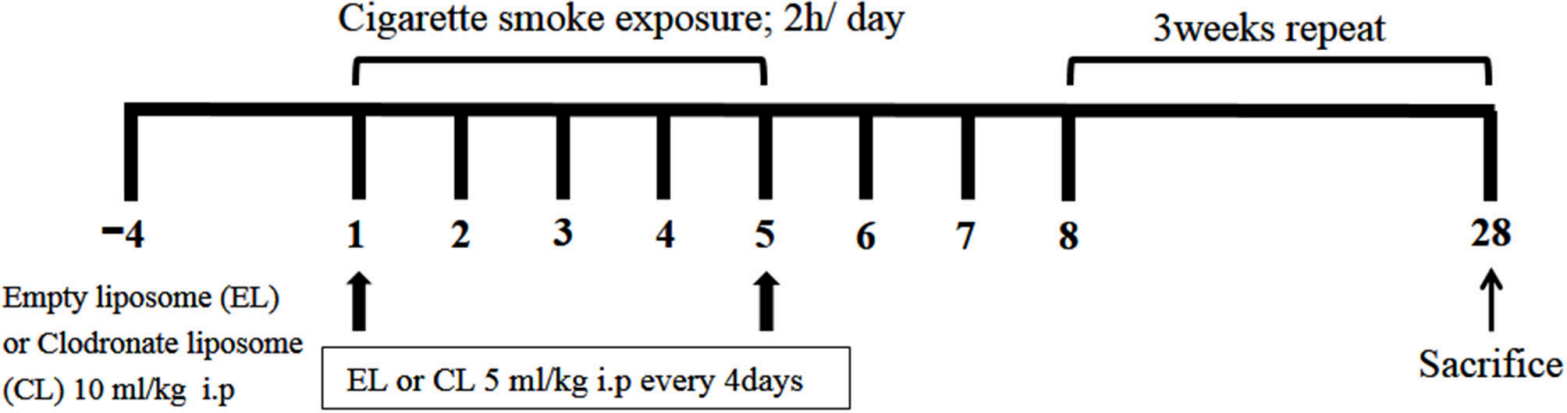
The schematic representation of the experimental procedure. C57BL/6 female mice were divided into 4 groups. 10 μl/kg of empty liposome (EL) or clodronate containing liposome (CL) was intraperitoneally injected 4 days before first cigarette smoke (CS) exposure, and 5 μl/kg of CL or EL was given by i.p. injection every 4 days during the experiment for further macrophage depletion. EL control group (*n* = 6) and CL control group (*n* = 6) mice were exposed to fresh air with distilled water (DW). EL CS group (*n* = 8) and CL CS group (*n* = 12) mice were subjected to whole-body CS exposure in a smoke chamber for four 30-min periods, between which they were rested for 30 min in a fresh air environment. It was repeated four times a day. This procedure was performed 5 days per week for 4 weeks in total.

All mice were divided into four groups: EL control group (*n* = 6) and CL control group (*n* = 6) mice were exposed to fresh air with distilled water (DW). EL CS group (*n* = 8), and CL CS group (*n* = 12) mice were exposed to CS (Reference Cigarette 3R4F without a filter, University of Kentucky, Lexington, KY, USA) with either DW. Mice in CS groups were subjected to whole-body exposure to CS in a smoke chamber for four 30 min periods with recovery in a fresh air environment for 30 min between each exposure for 5 days per week for 4 weeks. In our experiment, the mice were exposed to the side stream smoke, and each cigarette was completely burned out during the first 1 min due to the pressure generated from oil-less air pump (M-technology, Incheon, South Korea). In addition, the oil-less pump was set to the inhalation rate of 30 per 1 min. Individual body weights were measured twice a week, and the mice were sacrificed on day 28. These experiments were performed in triplicate. The experimental procedure of cigarette smoke exposure is described in Figure 1.

### Analysis of bronchoalveolar lavage fluid (BALF)

After the mice were sacrificed, PBS was infused into the lung and withdrawn via a cannula inserted into the trachea to collect bronchoalveolar lavage fluid (BALF). After three lavages, the cell pellet was resuspended in 1 ml of PBS for total cell and differential cell counts. Total cell concentrations were counted using a hemocytometer with Trypan blue stain, and the differential cell counts (neutrophils, macrophages and lymphocytes) were performed on slides prepared by cytocentrifugation and Diff-Quick staining (Life Technologies., Auckland, New Zealand) using light microscopy as we previously reported (Lim et al., 2017). The stained BAL cell slides were mounted with Canada balsam (Showa Chemical Co. Ltd., Tokyo, Japan). The stained BAL cell counts were done independently by 2 blinded investigators. Each evaluated the same BAL slides a second time, performing leukocyte differential counts in 5 microscopic fields, regardless of the total number of cells counted. Cells in a given area were not counted more than once. To detect cytokines and chemokine in BALF, BALF was centrifuged for 10 min at 300 g and 4°C and the supernatants were stored at −80°C for measurement of cytokines and chemokine. Levels of TNF-α, IL-6, IL-1β, and MCP-1 in the BALF were measured by enzyme-linked immunosorbent assay (ELISA) using a commercial kit (OptELATM Kits; BD Biosciences, San Diego, CA, USA) according to the manufacturer's protocols. The optical density was measured at 450 nm in a microplate reader (SoftMax Pro software; Sunnyvale, CA, USA). The optical densities obtained for TNF-α, IL-6, IL-1β, and MCP-1 were each divided by the total protein concentrations of the respective BAL fluid samples for standardization purposes. The total protein concentrations were determined by Bradford method using a Bio-Rad protein assay (Bio-Rad, Hercules, CA, USA) according to the manufacturer's protocol.

### Histological analysis

Lung and colon tissues were isolated from experimental animals and were fixed in 10% formaldehyde for at least 24 h. The specimens were embedded in paraffin, after which those were sectioned at 4 μm thickness using a rotary microtome for histological examination. Tissue paraffin sections were stained with hematoxylin and eosin solution (H&E, Sigma-Aldrich, MO, USA) to determine intensity of inflammation state. H&E-stained sections were evaluated using light microscopy at a magnification of 200×. The lung and colon sections were scored from 0 to 3 by investigators according to the following scoring characteristics in a blinded fashion: Lung index: Norma = 0, slight cell infiltration without emphysematous change = 1, moderate cell infiltration without emphysematous change = 2, moderate cell infiltration with emphysematous change = 3. Colon index: Norma = 0, slight cell infiltration without intestinal gland destruction = 1, moderate cell infiltration without intestinal gland destruction = 2, moderate cell infiltration with intestinal gland destruction = 3.

### Colonic tissue analysis

The whole colon was removed from the abdominal cavity, and the surrounding mesentery was removed and washed with PBS, and the colon weight and length were measured. Inside the colon was flushed with PBS using a gavage needle attached to a 10 mL syringe and the tissue was stored at −80°C for analysis. The colonic tissues were lysed by using T-PER tissue protein extraction reagent (Pierce, Rockford, IL, USA) containing a protease inhibitor cocktail (Roche, Indianapolis, IN, USA). The homogenate was centrifuged at 13,000 g at 4°C for 20 min. Protein concentrations were measured. Protein concentrations were measured using the method shown in section Analysis of Bronchoalveolar Lavage Fluid (BALF). The amount of TNF-α, IL-6, IL-1β, and MCP-1 in the colonic tissue was evaluated by ELISA using a commercial kit.

### Western blotting

MMPs protein expression in the lung and the colon tissues were examined by western blotting. The left lung and colon tissues were homogenized in the T-PER Tissue Protein Extraction Reagent (Pierce, Rockford, IL, USA) including protease inhibitor cocktail (Roche, Indianapolis, IN, USA), and the homogenates were centrifuged at 13,000 × g for 10 min at 4°C. Protein concentrations were measured using the method shown in section Analysis of Bronchoalveolar Lavage Fluid (BALF).

Proteins were diluted into SDS loading sample buffer and incubated at 95°C for 5 min, after which samples (20 μg/lane) were separated by SDS-polyacrylamide gel electrophoresis and transferred onto a nitrocellulose membrane. The transblotted membranes were blocked with 5% BSA buffer for 1 h at room temperature. The blocked membranes were incubated overnight at 4°C with the indicated primary antibodies: mouse β-actin (sc-8432), goat MMP-3 (sc-6839), mouse MMP-9 (sc-6840), and goat MMP-12 (sc-31809) (Santa Cruz Biotechnology, Dallas, TX, USA; diluted 1:1,000 in TBS-T). The blots were washed three times in TBS-T buffer, and then incubated with secondary antibodies. Blots were washed again, protein bands were visualized using an enhanced chemiluminescence western blot analysis system (AbClon Inc., Seoul, South Korea).

### Flow cytometry analysis

To examine the effect of CS exposure on the population of immune cells infiltrated in colonic LP. LP cells were separated into single cells using gentle MACS^TM^ dissociator. Single cells were stained to identify myeloid cells, CD4 T cells, CD8 T cells, B cells, macrophages, and dendritic cells respectively. Live leukocytes were identified based on CD45 gate. The following antibodies were used: CD11b-APC (17-0112-81, e-bioscience), CD4-FITC (53-0041-82, e-bioscience), CD8-APC (17-0081-81, e-bioscience), B220-PE (553089, BD Pharmingen), F4/80-PE (123110, BioLegend), CD11c-PE (12-0114-82, e-bioscience), and CD45-FITC (103108, BioLegend). The stained cells were detected by a FACSCalibur flow cytometer (Becton Dickinson, San Jose, CA, USA).

### Immunohistochemistry

Lung and colon tissues embedded in paraffin were sectioned and rehydrated. The tissues were antigen retrieved in tri-sodium citrate buffer containing 0.2% tween 20 (pH 6.0) with an autoclave for 1 min. 3% H_2_O_2_ solution was treated for 10 min to block endogenous peroxidase activity. The tissues were washed in PBS and blocked with 1.5% bovine serum albumin (BSA) containing 0.2% triton X-100 for 1h at room temperature. Rat anti-mouse CD11b primary antibodies (MCA74G) (Abd Serotec; 1:200) and biotinylated anti-Rat antibodies (BA-9401) (Vector laboratories; 1:1,000) were diluted in 0.5% BSA. The slides were overnight incubated with primary antibodies at 4°C and incubated with secondary antibodies for 1h at room temperature. The avidin-biotin complex kit (Vectastain ABC kit; PK-6102) (Vector Laboratories, Burlingame, CA, USA) and diamminobenzidine-HCl (DAB) kit (SK-4100) (Vector Laboratories, Burlingame, CA, USA) were used to visualize the tissue. The slides were mounted and observed under the microscope.

### Statistical analysis

Statistical analysis of the data was carried out using Prism 4 software (GraphPad Software Inc., CA, USA). Data are presented as mean ± standard error of the mean (SEM). The statistical significance analysis was calculated using two-tailed Student's *t*-test for single comparisons or two-way ANOVA or two-way ANOVA followed by Bonferroni *post-hoc*. Results with *p* < 0.05 were considered statistically significant.

## Results

### Macrophage depletion ameliorated body weight loss caused by cigarette smoke

**Figure 2:**
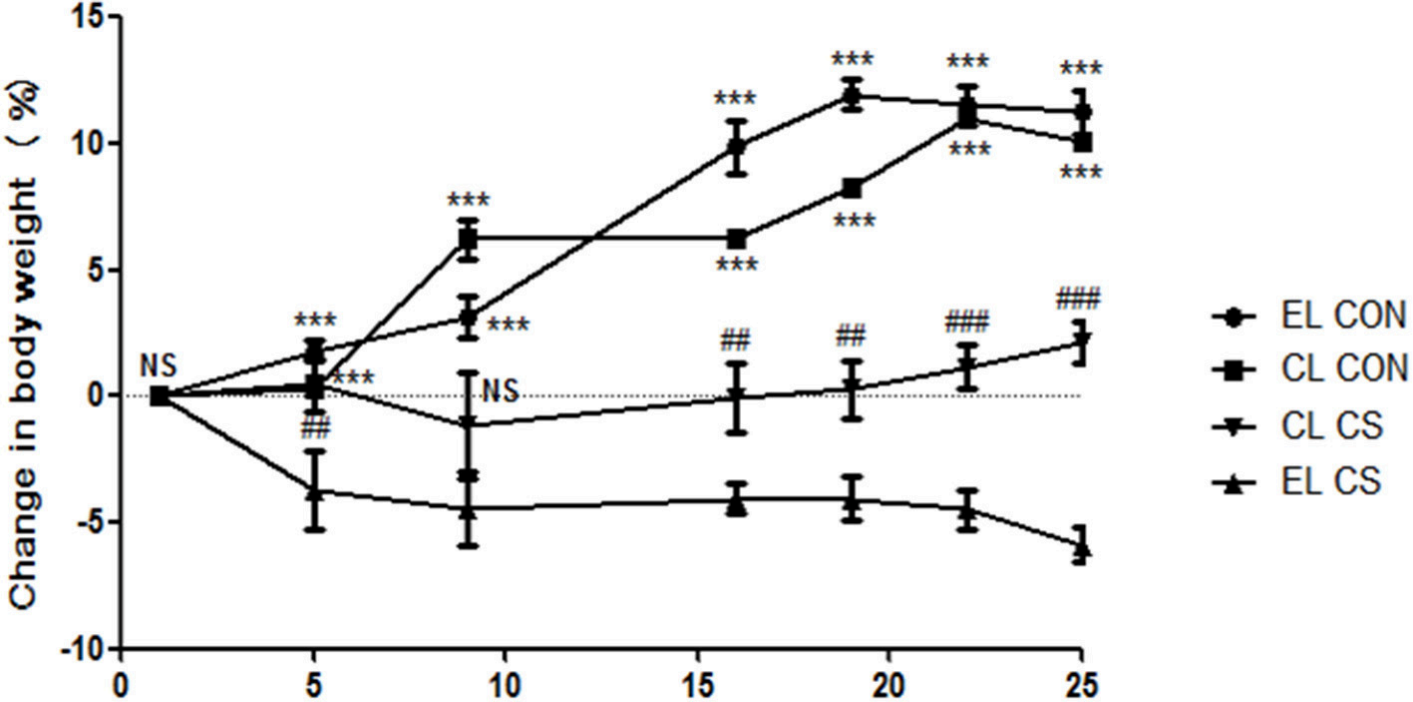
The effect of macrophage depletion on CS exposure-induced body weight loss. Body weights were measured for each group of mice twice a week. EL CON (*n* = 6): control mice with empty liposome; EL CS (*n* = 8): CS-exposed mice with empty liposome; CL CON (*n* = 6: control mice with clodronate containing liposome; CL CS (*n* = 12): CS-exposed mice with clodronate containing liposome. Values are expressed as mean ± SEM. Statistical analysis was performed by a two-way ANOVA followed by Bonferroni *post-hoc*; ^***^p < 0.001 indicated a significant difference vs. fresh air group. ^*##*^*p* < 0.01 and ^*###*^*p* < 0.001 indicated a significant difference vs. empty liposome group.

Body weight loss is a typical feature of pathological change in COPD and CD (Wack and Rodin, 1982; Muers and Green, 1993; Torres et al., 2017). As shown in Figure 2, the body weights of CON group that were administered with empty liposme (EL CON) increased from 17.1 ± 0.5 to 19 ± 0.5 g (11.2% increase) over 4 weeks. Similarly, the body weights of CON group treated with clodronate liposome (CL CON) increased from 17.2 ± 0.5 to 18.9 ± 0.5 g (10% increase). In comparison, the body weights of EL CS group decreased 5% compared to those of EL CON group (^***^p < 0.001). Compared to the body weights of CS-exposed mice with empty liposome (EL CS), those of the clodronate liposome administered CS group (CL CS) increased 2.1% over 4 weeks (^*###*^*p* < 0.001).

### Macrophage depletion inhibited the inflammatory cells infiltration and histological changes in lung

**Figure 3:**
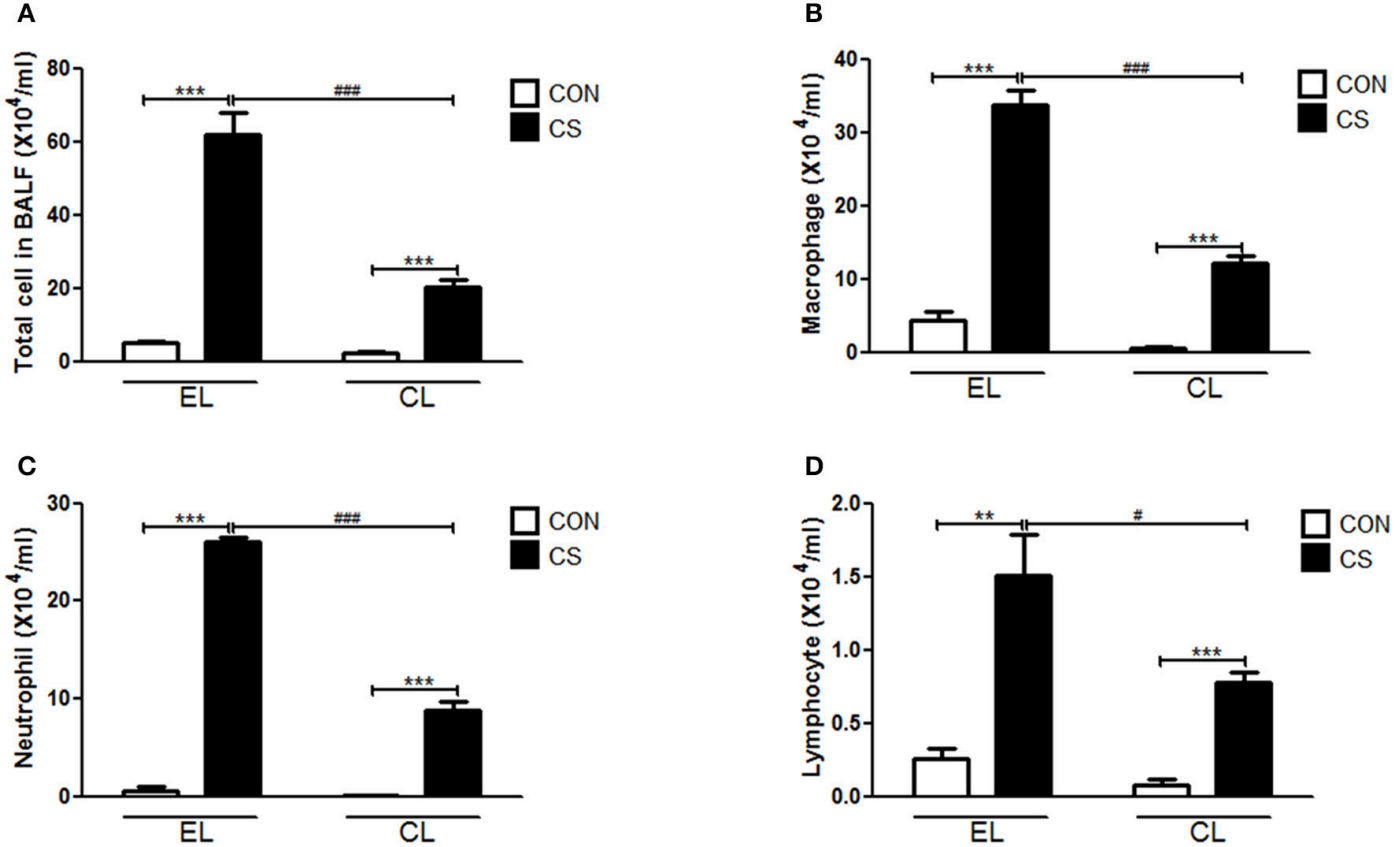
The effect of macrophage depletion on CS exposure-induced immune cell. infiltration in bronchoalveolar lavage fluid (BALF). The number of inflammatory cells was confirmed 4 weeks after CS exposure. Total cells **(A)**, macrophages **(B)**, neutrophils **(C)**, and lymphocytes **(D)** in BALF were counted with light microscopy after Diff-Quick staining. EL CON (*n* = 6): control mice with empty liposome; EL CS (*n* = 8): CS-exposed mice with empty liposome; CL CON(*n* = 6): control mice with clodronate containing liposome; CL CS (*n* = 12): CS-exposed mice with clodronate containing liposome. Values are expressed as mean ± SEM. Statistical analysis was performed by using a two-way ANOVA; ^**^p < 0.01 and ^***^p < 0.001 indicated a significant difference vs. fresh air group. ^#^*p* < 0.05 and ^*###*^*p* < 0.001 indicated a significant difference vs. empty liposome group.

**Figure 4:**
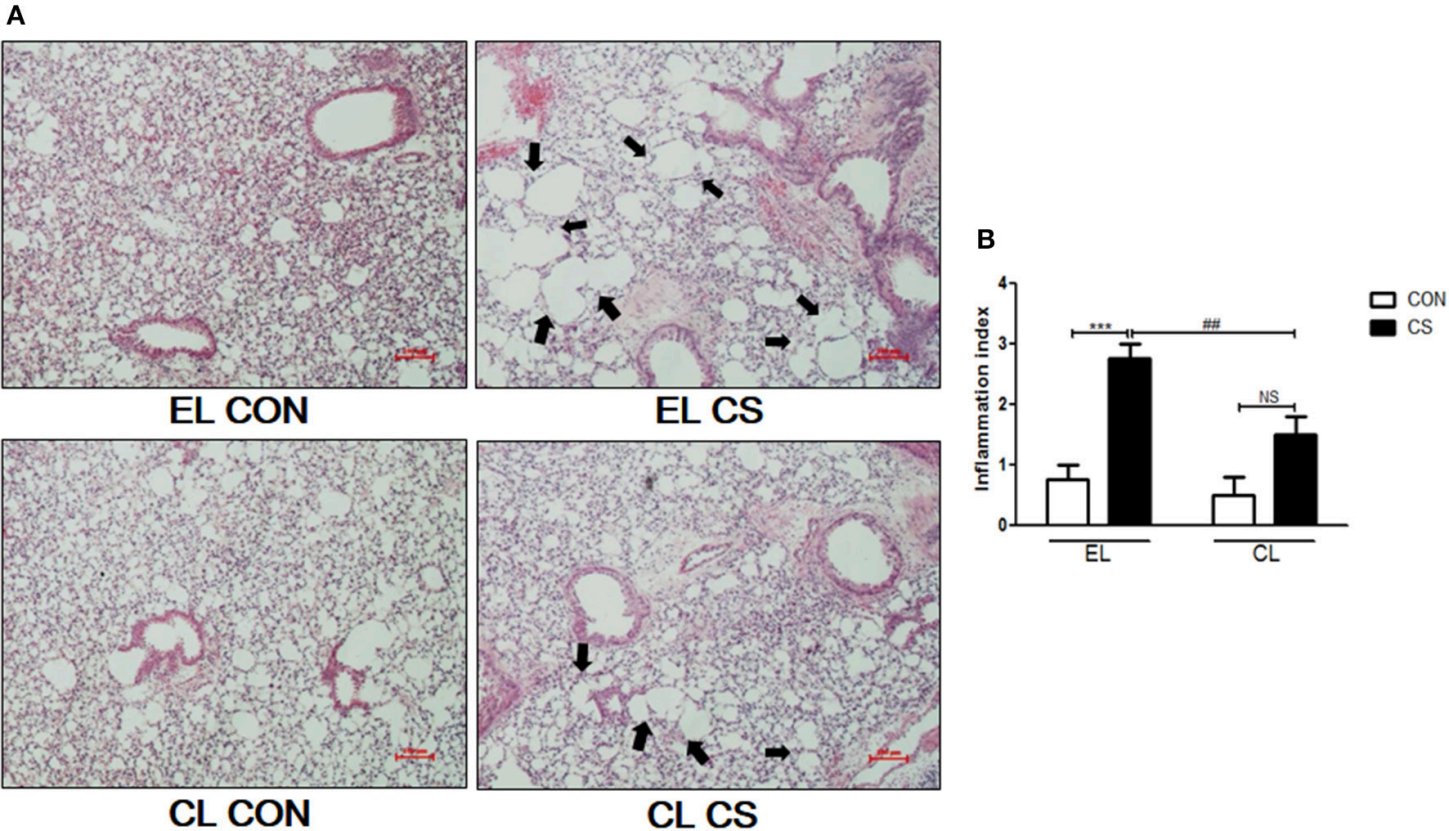
The effect of macrophage depletion on CS exposure-induced histological changes in the lung. Fixed lung tissues were sectioned at 4 μm, and stained with hematoxylin and eosin (H&E) solution **(A)**. The lung inflammation scores were represented according to the degree of emphysematous changes **(B)**. Shown by arrows are the emphysematous changes. EL CON (*n* = 6): control mice with empty liposome; EL CS (*n* = 8): CS-exposed mice with empty liposome; CL CON (*n* = 6): control mice with clodronate containing liposome; CL CS (*n* = 12): CS-exposed mice with clodronate containing liposome. Values are expressed as mean ± SEM. Statistical analysis was performed by using a two-way ANOVA; ^***^p < 0.001 indicated a significant difference vs. fresh air group. ^*##*^*p* < 0.01 indicated a significant difference vs. empty liposome group. NS indicated not significant difference. Magnification × 200.

The inflammatory cell infiltration in bronchoalveolar lavage fluid (BALF) was evaluated. EL CS group showed a significant increase in the number of total cells (^***^p < 0.001), macrophages (^***^p < 0.001), neutrophils (^***^p < 0.001), and lymphocytes (^**^p < 0.01) compared to the EL CON group respectively. Among all the inflammatory cells counted, macrophages accounted for over half of the total. In comparison, CL CS group significantly reduced the amount of total cells (^*###*^*p* < 0.001), macrophages (^*###*^*p* < 0.001), neutrophils (^*###*^*p* < 0.001), and lymphocytes (^#^*p* < 0.05) compare with the EL CS group (Figure 3 and Supplementary Figure 1). In addition, histologic examination was performed by H&E staining to investigate the effect of macrophage depletion in pathologic change of lung from CS exposure. Compared to the EL CON group, EL CS group showed significant emphysematous changes with the perivascular and peribronchial cell accumulation (^***^p < 0.001). However, compared to the CL CON group, CL CS group showed no significant emphysematous changes with the perivascular and peribronchial cell accumulation. In contrast, CL CS group showed significantly attenuated the emphysematous changes and the cell accumulation into regions surrounding the bronchi of CS-exposed mice compared to the EL CS group (##*p* < 0.01) (Figure 4). These results provided evidence that CS inhalation induces lung inflammation, and clodronate-liposome-induced macrophage depletion ameliorates such inflammatory response.

### Macrophage depletion attenuated macroscopic and histopathological changes in the colon of CS-exposed mice

**Figure 5:**
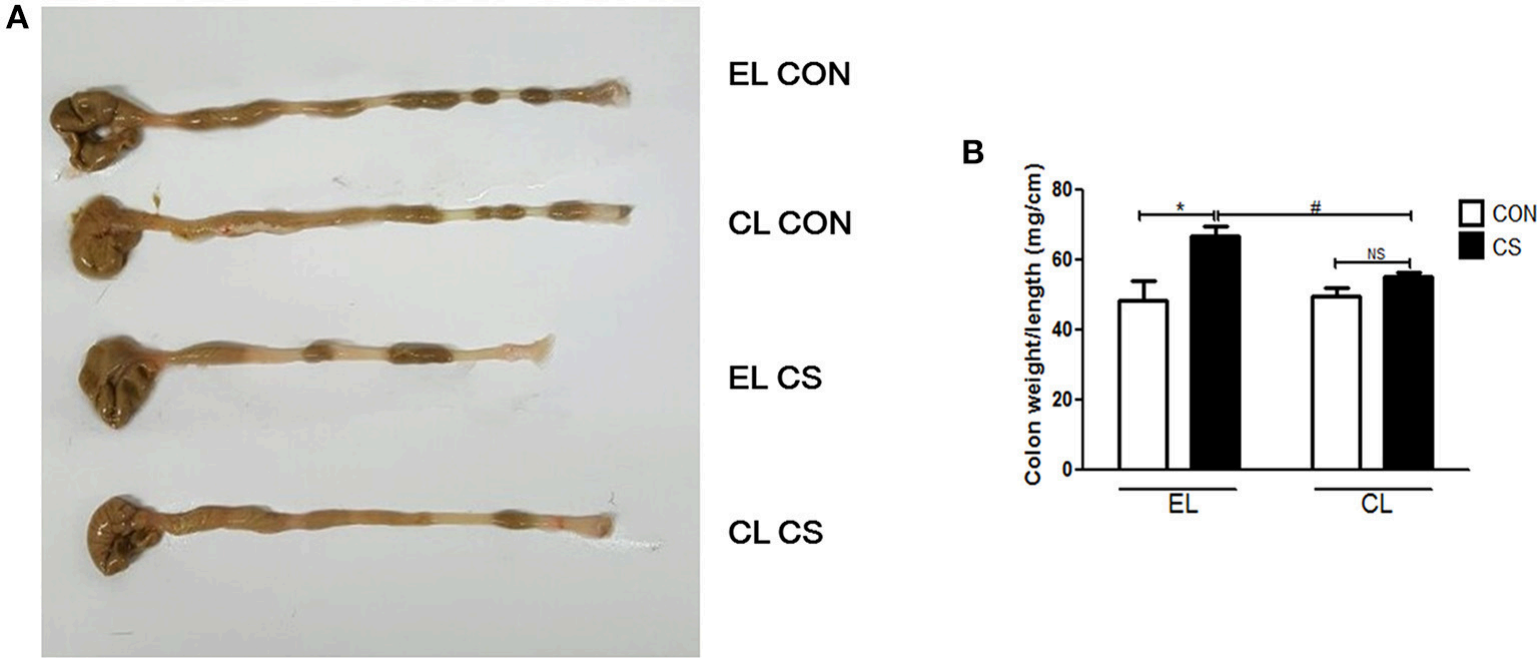
The effect of macrophage depletion on CS exposure-induced macroscopic changes in the colon. Colonic inflammation in mice was induced by repeated CS exposure. The colon length **(A)** was evaluated by a macroscopic examination with a ruler, and the weight was measured after removing the surrounding mesentery with PBS. Weight/length ratio **(B)** as a marker of colitis was depicted in a graph. EL CON (*n* = 6): control mice with empty liposome; EL CS (*n* = 8): CS-exposed mice with empty liposome; CL CON (*n* = 6): control mice with clodronate containing liposome; CL CS (*n* = 12): CS-exposed mice with clodronate containing liposome. Values are expressed as mean ± SEM. Statistical analysis was performed by using a two-way ANOVA; ^*^p < 0.05 indicated a significant difference vs. fresh air group. ^#^*p* < 0.05 indicated a significant difference vs. empty liposome group. NS indicated not significant difference.

**Figure 6:**
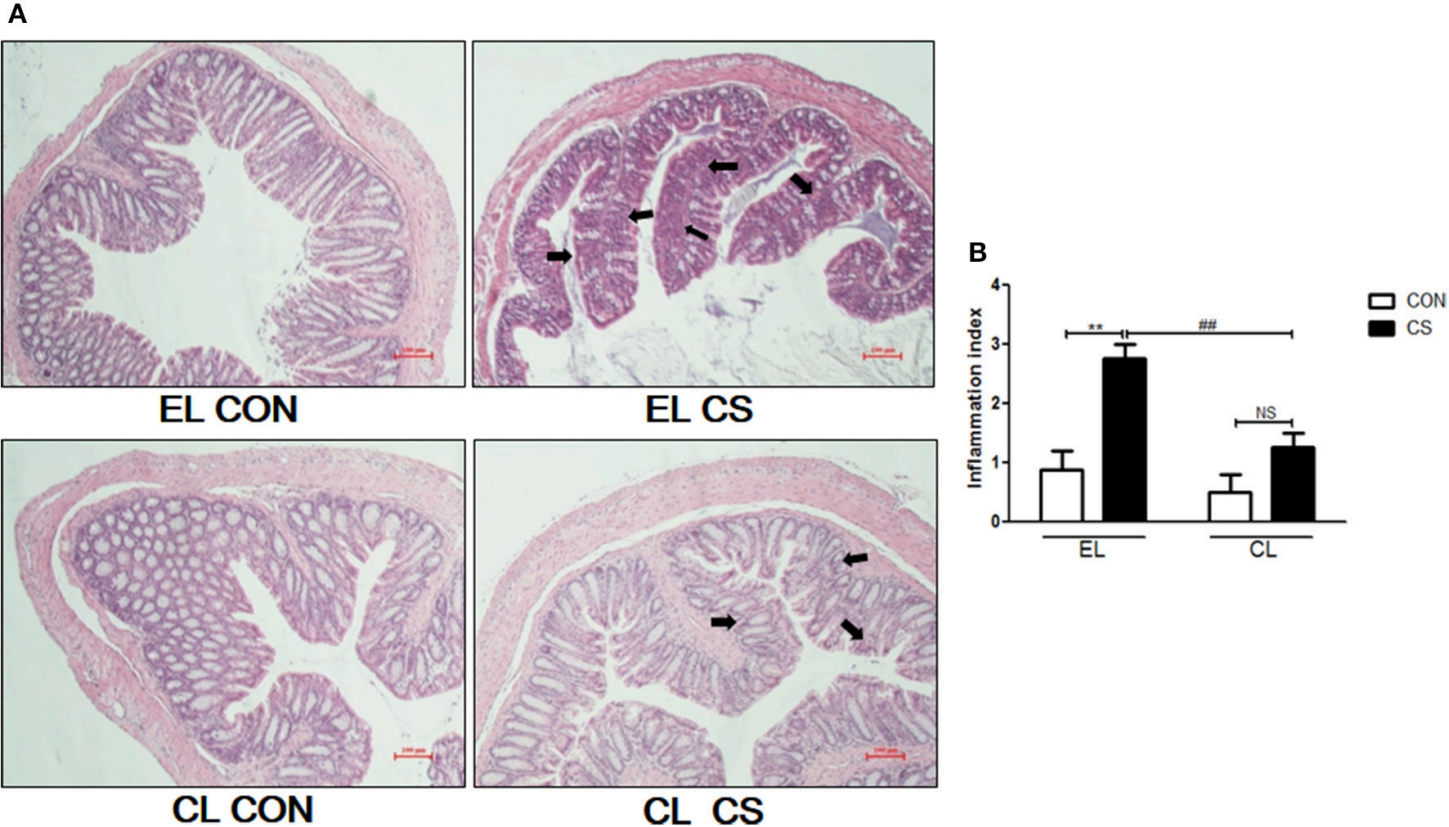
The effect of macrophage depletion on CS exposure-induced histopathological changes in the colon. Fixed colon tissues were sectioned at 4 μm, and stained with H&E solution **(A)**. The inflammation index of colon was depicted by a blind test as the graph **(B)**. Shown by arrows are the mucosal damage from cell infiltration. EL CON(*n* = 6): control mice with empty liposome; EL CS (*n* = 8): CS-exposed mice with empty liposome; CL CON (*n* = 6): control mice with clodronate containing liposome; CL CS (*n* = 12): CS-exposed mice with clodronate containing liposome. Values are expressed as mean ± SEM. Statistical analysis was by a two-way ANOVA; ^**^p < 0.01 indicated a significant difference vs. fresh air group. ^*##*^*p* < 0.01 indicated a significant difference vs. empty liposome group. NS indicated not significant difference. Magnification × 200.

Colon length shortening and colon weight increase from inflammatory cell infiltration are common phenomena of colonic inflammation. Colon weight/length ratio was widely used as the indicator for the disease-related inflammation intensity (Whittem et al., 2010; Lin et al., 2015). Thus, colonic inflammation index was evaluated by measuring the weight/length ratio of the colon. Compared with EL CON and CL CON groups, EL CS group showed the colon length shortening. Also EL CS group showed the significant increase in weight/length ratio (^*^p < 0.05) compared to EL CON group, while no significance was shown when compared to CL CON group. In contrast, CL CS group significantly reduced the colon weight/length ratio compared to EL CS group (^#^*p* < 0.05) (Figure 5). These results demonstrated that CS exposure induced colon length shortening and intestinal wall thickening, and macrophage depletion reduced such phenomena. Moreover, our histologic study data supported these results. Histologic examination was performed by H&E staining to examine the effect of macrophage depletion in pathologic change of colon from CS exposure. EL CS group showed a distinct mucosal damage with cell infiltration compared with the EL CON group (^**^p < 0.01). However, CL CS group mice showed only a slight mucosal damage with cell infiltration compared with CL CON group. In contrast, CL CS group showed significantly attenuated the mucosal damage with cell infiltration compared to the EL CS group (^*##*^*p* < 0.01) (Figure 6). These results provided further evidence that CS exposure provokes colonic inflammation, and clodronate injection attenuates such inflammatory response.

### Macrophage depletion decreased inflammatory cytokines and chemokine in BALF and the colon

**Figure 7:**
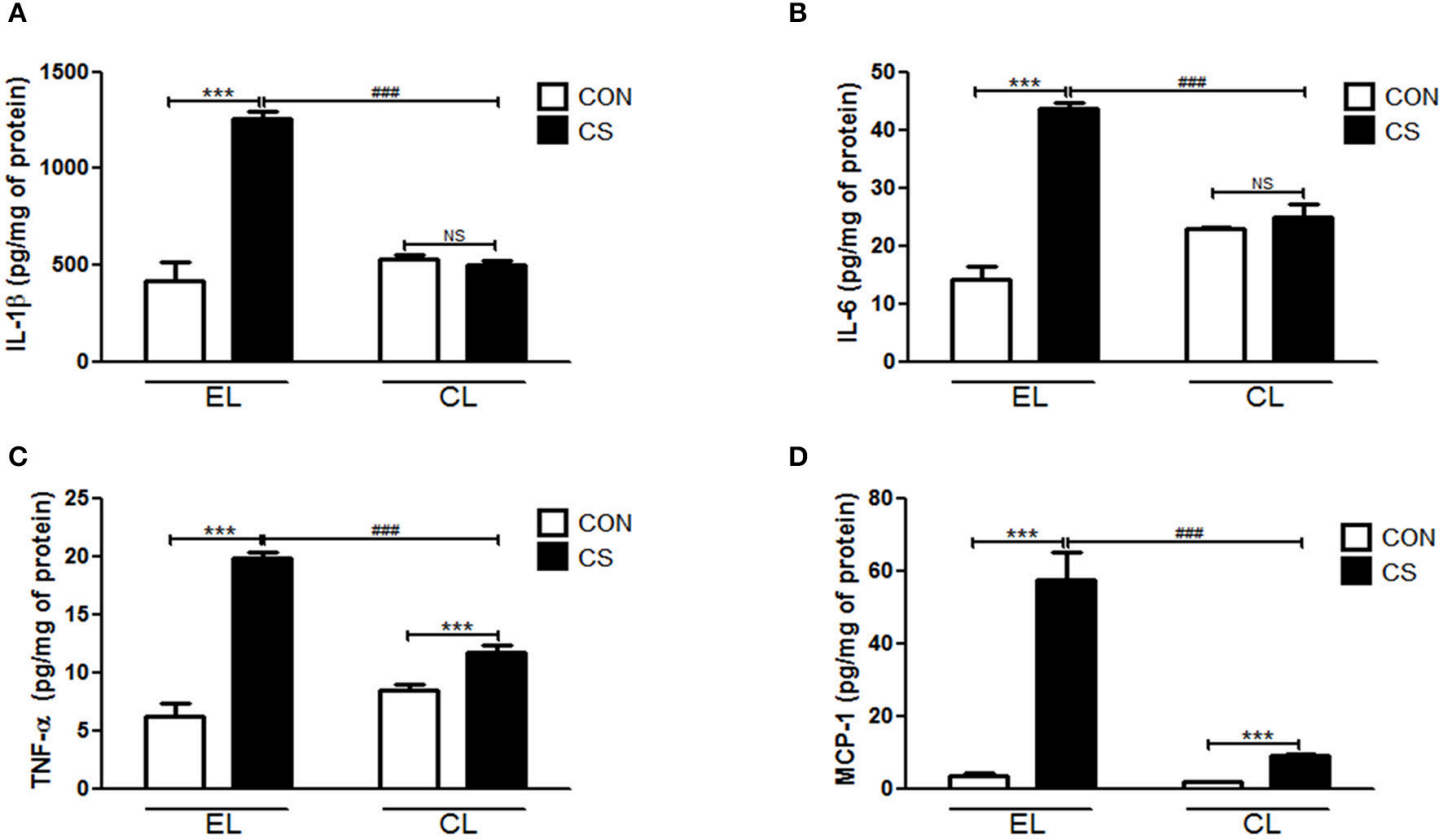
Effects of macrophage depletion on the CS exposure-induced expressions of inflammatory cytokines and chemokine in BALF. The amount of IL-1β **(A)**, IL-6 **(B)**, TNF-α **(C)**, and MCP-1 **(D)** in the BALF was measured by enzyme-linked immunosorbent assay (ELISA). EL CON (*n* = 6): control mice with empty liposome; EL CS (*n* = 8): CS-exposed mice with empty liposome; CL CON (*n* = 6): control mice with clodronate containing liposome; CL CS (*n* = 12): CS-exposed mice with clodronate containing liposome. Values are expressed as mean ± SEM. Statistical analysis was performed by using a two-way ANOVA; ^***^p < 0.001 indicated a significant difference vs. fresh air group. ^*###*^*p* < 0.001 indicated a significant difference vs. empty liposome group. NS indicated not significant difference.

**Figure 8:**
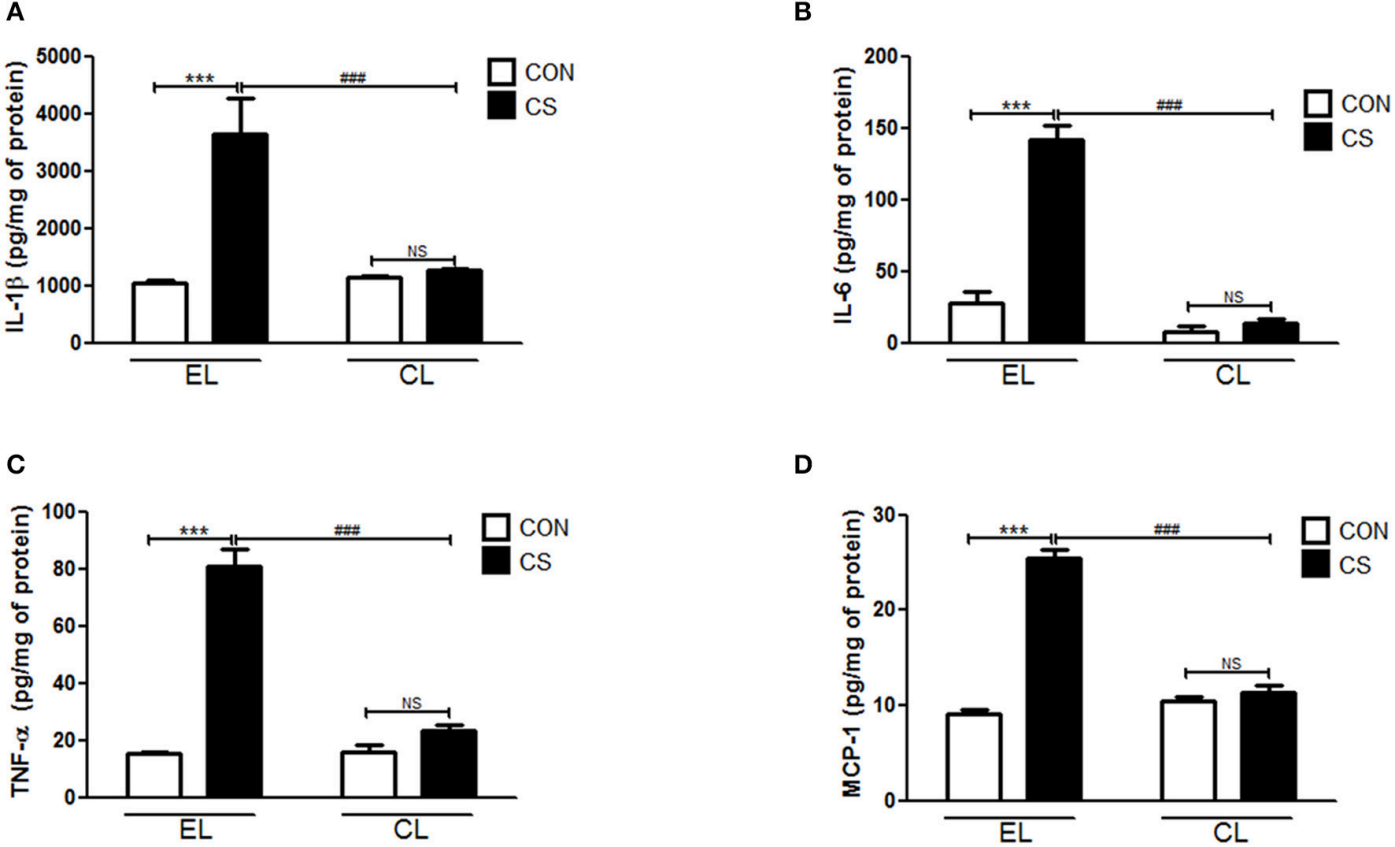
The effect of macrophage depletion on CS exposure-induced change in the expression of inflammatory cytokines and chemokine in the colonic tissue. The colonic tissues were lysed by using T-PER tissue protein extraction reagent (Pierce, Rockford, IL, USA) containing a protease inhibitor cocktail (Roche, Indianapolis, IN, USA). Protein concentrations were measured by using the Bradford method. The amount of IL-1β **(A)**, IL-6 **(B)**, TNF-α **(C)**, and MCP-1 **(D)** were measured by ELISA. EL CON (*n* = 6): control mice with empty liposome; EL CS (*n* = 8): CS-exposed mice with empty liposome; CL CON (*n* = 6): control mice with clodronate containing liposome; CL CS (*n* = 12): CS-exposed mice with clodronate containing liposome. Values are expressed as mean ± SEM. Statistical analysis was performed by using a two-way ANOVA; ^***^p < 0.001 indicated a significant difference vs. fresh air group. ^*###*^*p* < 0.001 indicated a significant difference vs. empty liposome group. NS indicated not significant difference.

The amount of inflammatory cytokines and chemokine were measured by using ELISA. Compared to the EL CON group, EL CS group showed the significant increase in the productions of IL-1β (^***^p < 0.001), IL-6 (^***^p < 0.001), TNF-α (^***^p < 0.001), and MCP-1(^***^p < 0.001) in BALF respectively. In contrast, CL CS group showed significant reduction in IL-1β (^*###*^*p* < 0.001), IL-6 (^*###*^*p* < 0.001), TNF-α (^*###*^*p* < 0.001 and MCP-1 (^*###*^*p* < 0.001) in BALF respectively compared to the EL CS group (Figure 7). The assay performed with colon tissue protein showed similar results. The production of these all tested inflammatory cytokines and chemokine were significantly increased in EL CS group compared to the EL CON group (^***^p < 0.001). However, when compared to CL CON group, CL CS group showed no significance. Moreover, compared with EL CS group, the amount of IL-1β (^*###*^*p* < 0.001), IL-6 (^*###*^*p* < 0.001), TNF-α (^*###*^*p* < 0.001), and MCP-1 (^*###*^*p* < 0.001) were significantly reduced in CL CS group respectively (Figure 8). These results demonstrated that CS induces the lung and large intestine inflammation, and clodronate-liposome-induced macrophage depletion inhibits these inflammatory responses.

### Macrophage depletion inhibited MMPs expressions induced by CS exposure

**Figure 9:**
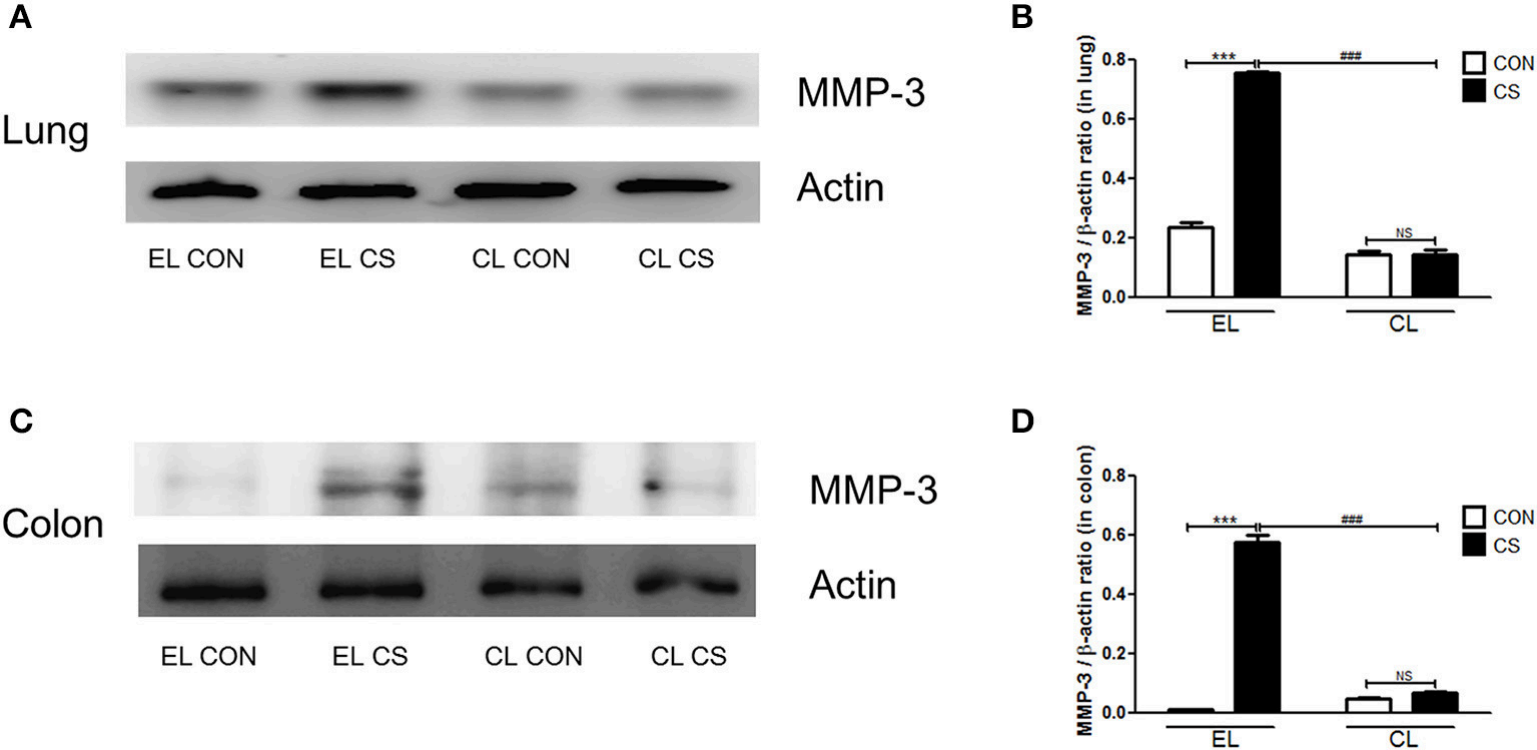
The effect of macrophage depletion on CS exposure-induced changes in MMP-3 expression in the lung and colon. Proteins in the lung and colon were extracted by using T-PER buffer containing protease inhibitor cocktail, and the total protein concentration was measured using Bradford assay. MMP-3 expression in the lung **(A)** and colon **(C)** were evaluated by western blotting. Relative MMP-3 levels in the lung **(B)** and colon **(D)** were quantified using the Image J software, and normalized to the levels of β-actin. EL CON (*n* = 6): control mice with empty liposome; EL CS (*n* = 7): CS-exposed mice with empty liposome; CL CON (*n* = 6): control mice with clodronate containing liposome; CL CS (*n* = 9): CS-exposed mice with clodronate containing liposome. Values are expressed as mean ± SEM. Statistical analysis was performed by using a two-way ANOVA; ^***^p < 0.001 indicated a significant difference vs. fresh air group. ^*###*^*p* < 0.001 indicated a significant difference vs. empty liposome group. NS indicated not significant difference.

**Figure 10:**
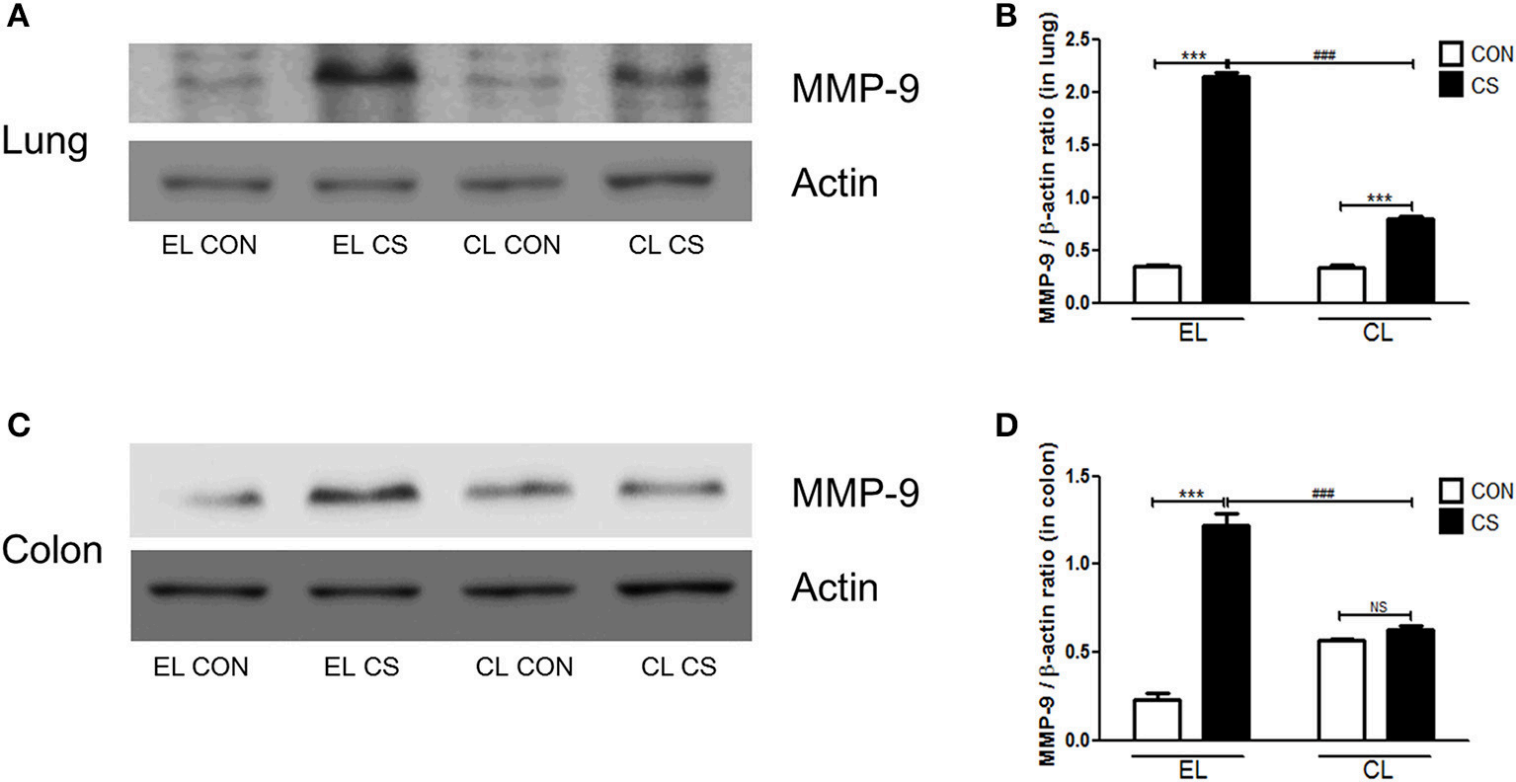
The effect of macrophage depletion on CS exposure-induced changes in MMP-9 expression in the lung and colon. Proteins in the lung and colon were extracted by using T-PER buffer containing protease inhibitor cocktail, and the total protein concentration was measured using Bradford assay. MMP-9 expressions in the lung **(A)** and the colon **(C)** were examined by western blotting. Relative MMP-9 levels in the lung **(B)** and the colon **(D)** were quantified using the ImageJ software, and normalized to the levels of β-actin. EL CON (*n* = 6): control mice with empty liposome; EL CS (*n* = 7): CS-exposed mice with empty liposome; CL CON (*n* = 6): control mice with clodronate containing liposome; CL CS (*n* = 9): CS-exposed mice with clodronate containing liposome. Values are expressed as mean ± SEM. Statistical analysis was performed by using a two-way ANOVA; ^***^p < 0.001 indicated a significant difference vs. fresh air group. ^*###*^*p* < 0.001 indicated a significant difference vs. empty liposome group. NS indicated not significant difference.

**Figure 11:**
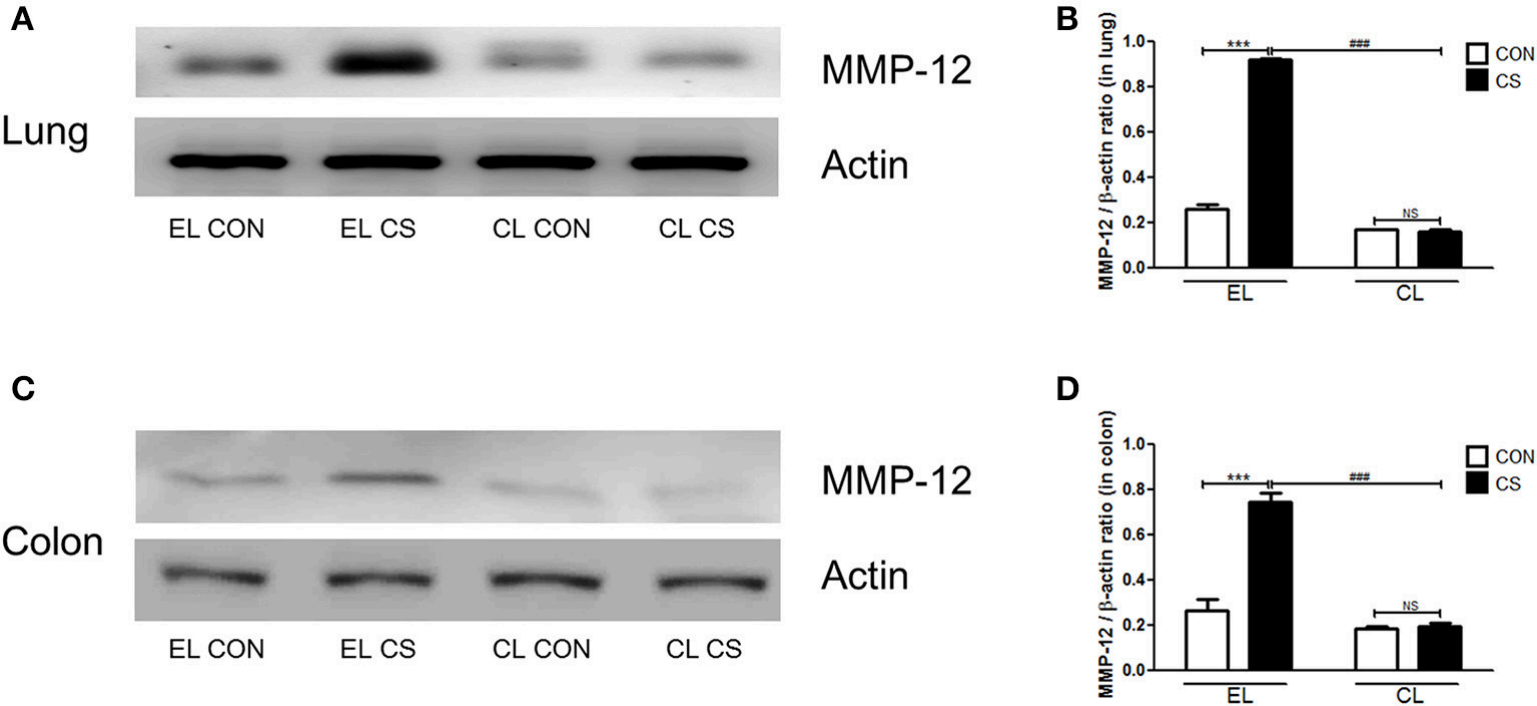
The effect of macrophage depletion on the CS exposure-induced changes in MMP-12 expression in the lung and colon. Proteins in the lung and the colon were extracted by using T-PER buffer containing protease inhibitor cocktail, and the total protein concentration was measured using Bradford assay. MMP-12 expressions in the lung **(A)** and the colon **(C)** were analyzed by western blotting. Relative MMP-12 levels in the lung **(B)** and the colon **(D)** were quantified using the ImageJ software, and normalized to the levels of β-actin. EL CON (*n* = 6): control mice with empty liposome; EL CS (*n* = 7): CS-exposed mice with empty liposome; CL CON (*n* = 6): control mice with clodronate containing liposome; CL CS (*n* = 9): CS-exposed mice with clodronate containing liposome. Values are expressed as mean ± SEM. Statistical analysis was performed by using a two-way ANOVA; ^***^p < 0.001 indicated a significant difference vs. fresh air group. ^*###*^*p* < 0.001 indicated a significant difference vs. empty liposome group. NS indicated not significant difference.

The effect of macrophage depletion on MMPs protein expressions in the lung and colon of CS-exposed mice model were evaluated by western blot analysis. Compared to the EL CON group, EL CS group showed a significant increase in MMP-3, MMP-9, and MMP-12 expression in the lung and colon tissues (^***^p < 0.001) respectively. In contrast, CL CS group showed a significant increase in the expression MMP-9 (^***^p < 0.001) compared to the CL CON group. However, EL CS group did not show significant inductions in the expressions of MMP-3 and MMP-12 compared to the EL CON group. But, compared with the EL CS group, CL CS group showed significant downregulation of the expressions of MMP-3 (^*###*^*p* < 0.001), MMP-9 (^*###*^*p* < 0.001), and MMP-12 (^*###*^*p* < 0.001) respectively. Similar result was shown in the colon. Compared to the CL CON group, CL CS group did not induce significantly the expressions of MMP-3, MMP-9, and MMP-12. However, compared with the EL CS group, CL CS group showed significant downregulation of the expressions of MMP-3 (^*###*^*p* < 0.001), MMP-9 (^*###*^*p* < 0.001), and MMP-12 (^*###*^*p* < 0.001) respectively (Figures 9–11). These results provided further evidence that CS exposure causes inflammation response in the lung and colon, and that macrophage depletion reduces the expression of inflammatory mediators in both tissues.

### Treatment of clodronate liposome successfully depleted macrophages from mice tissue

**Figure 12:**
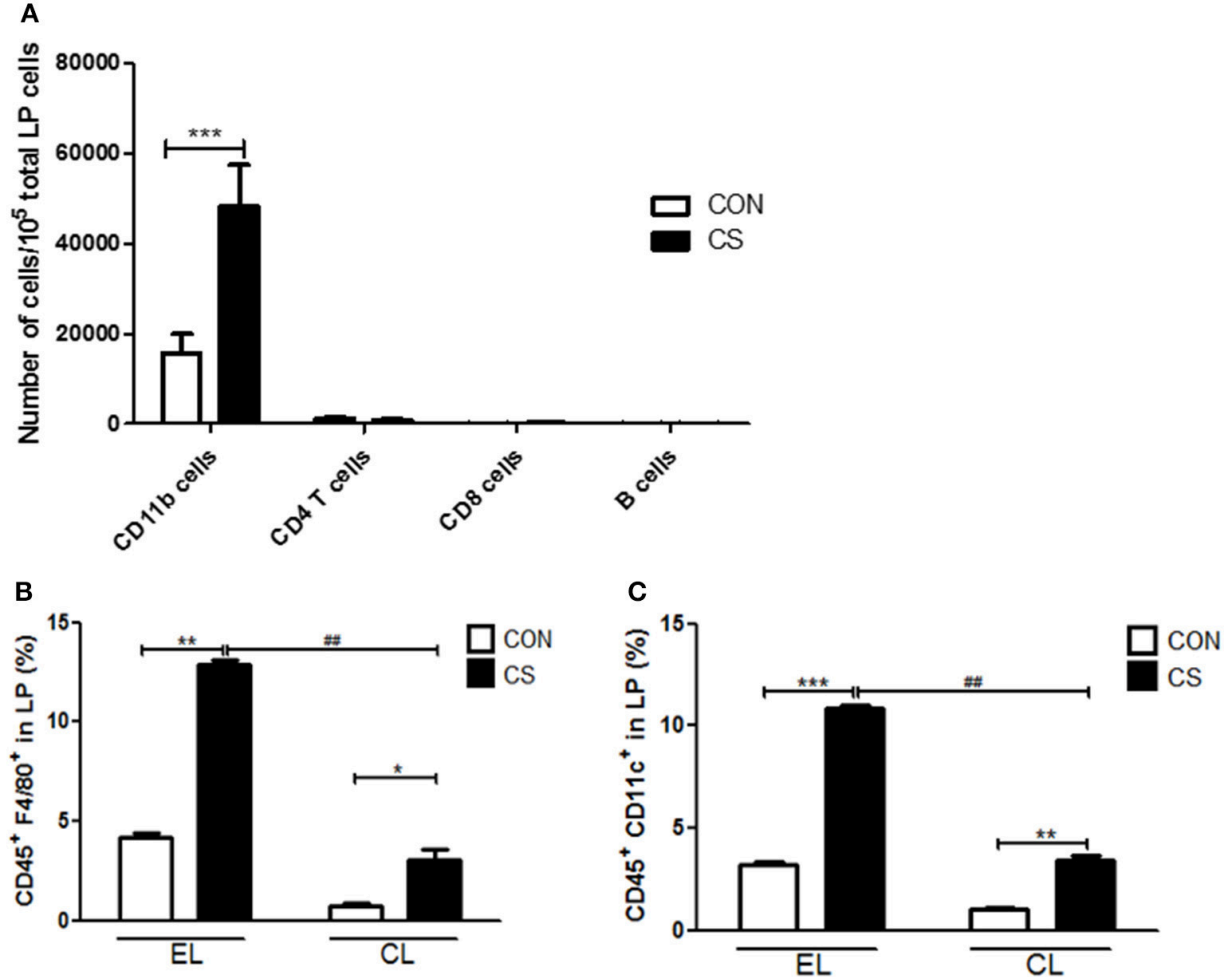
The effect of CS exposure on immune cell populations in the lamina propria. The infiltrated cell populations of CD11b cells, CD4 T cells, CD8 T cells and B cells in the LP were counted by flow cytometry (*n* = 4) **(A)**. The populations of CD45^+^F4/80^+^ macrophages **(B)** and CD45^+^CD11c^+^ dendritic cells **(C)** in LP were analyzed by flow cytometry. EL CON (*n* = 6): control mice with empty liposome; EL CS(*n* = 8): CS-exposed mice with empty liposome; CL CON (*n* = 6): control mice with clodronate containing liposome; CL CS(*n* = 10): CS-exposed mice with clodronate containing liposome. Values are expressed as mean ± SEM. Statistical analysis was performed by using a two-way ANOVA; ^*^p < 0.05, ^**^p < 0.01, and ^***^p < 0.001 indicated a significant difference vs. fresh air group. ^*##*^*p* < 0.01 indicated a significant difference vs. empty liposome group.

In order to investigate the increased inflammatory cell infiltration into colonic LP by CS exposure, the population of total CD11b myeloid cells including macrophages and dendritic cells, CD4 T cells, CD8 T cells, and B cells from LP, were measured by flow cytometry. The major population of LP-infiltrating cells was CD11b^+^ myeloid cells. CD11b^+^ cells were significantly increased by CS exposure compared to control(^***^p < 0.001). However, there was no significant differences between control and CS group in the population of CD4 T cells, CD8 T cells and B cells in colonic LP (Figure 12A).

Next, the populations of F4/80^+^ macrophages and CD11c^+^ dendritic cells (DCs) in LP were analyzed. EL CS group showed a significant increase in macrophages and DCs compared to EL CON group (^**^p < 0.01 and ^***^p < 0.001), and CL CS group showed a significant increase in macrophages and DCs compared to CL CON group (^*^p < 0.05 and ^**^p < 0.01). Although macrophage and DC populations were increased by CS exposure in clodronate liposome-treated groups, the infiltration of macrophages and dendritic cells into LP was significantly lower in CL CS group than that in EL CS group respectively (^*##*^*p* < 0.01 and ^*##*^*p* < 0.01), (Figures 12B,C).

**Figure 13:**
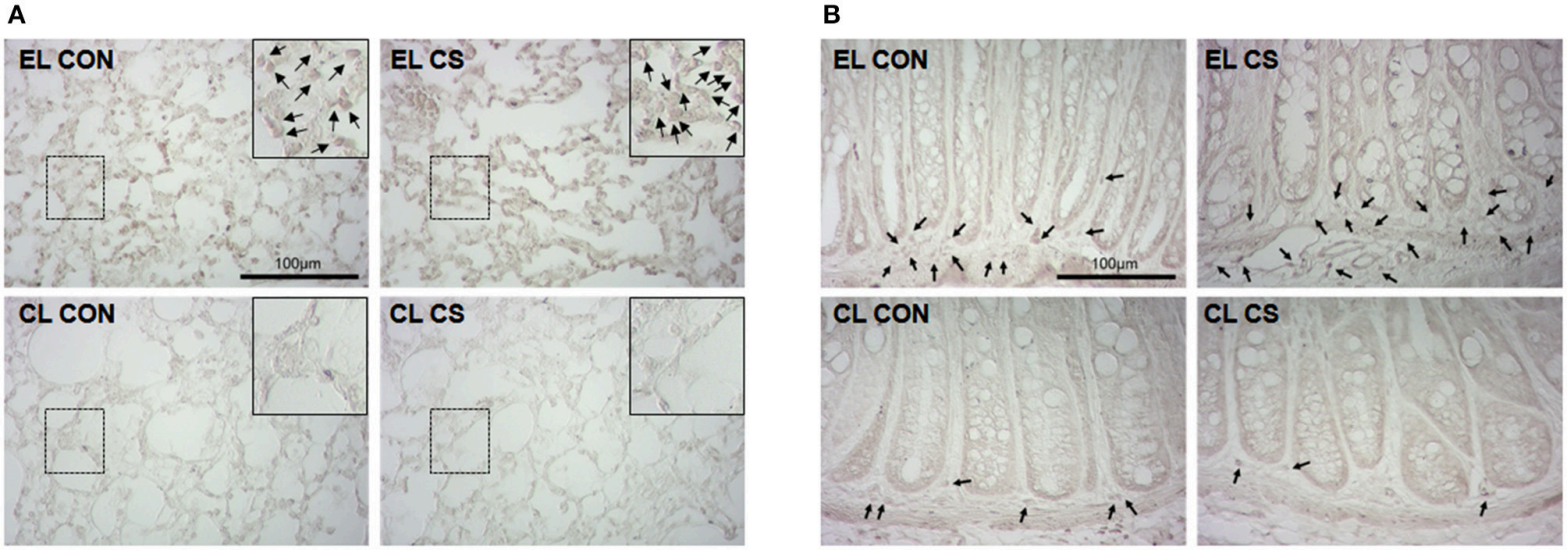
Depletion of macrophages by clodronate liposome. The CD11b^+^ macrophages in lung **(A)** and colon **(B)** tissue sections were detected by immunohistochemistry. EL CON *(n* = 6): control mice with empty liposome; EL CS (*n* = 8): CS-exposed mice with empty liposome; CL CON (*n* = 6): control mice with clodronate containing liposome; CL CS (*n* = 10): CS-exposed mice with clodronate containing liposome. The macrophages were marked with black arrow heads. Magnification × 400. Scale bars 100 μm.

To verify the depletion of macrophages induced by clodronate liposome, CD11b^+^ cells were visualized with DAB staining in lung and colon tissues. The lungs and colons of EL treated CS group showed increased number of CD11b^+^ cells compared to EL treated fresh air group. However, the number of CD11b^+^ cells in the lungs and colons from CL treated CS-exposed mice showed no significant difference compared to CL treated fresh air group. In addition, very few number of CD11b^+^ cells were detected in lung and colonic LP from CL treated mice (Figure 13). These results showed that macrophages were effectively depleted by clodronate liposome in the lung and colonic LP.

## Discussion

Our previous study demonstrated that repeated cigarette smoke (CS) exposure provokes inflammation in the lung and colon together, and noted the current body of scholarship showing the link between these two organs (Lim et al., 2017). More importantly, we confirmed from the study that macrophages and inflammatory mediators from macrophage including cytokines, chemokine, and proteases are closely related with the inflammatory response in the lung and colon (Lim et al., 2015, 2017). Several studies reported that activated macrophages release inflammatory mediators, contributing to inflammatory progression and tissue destruction in respiratory diseases such as chronic obstructive pulmonary disease (COPD), asthma, emphysema, and pulmonary fibrosis (Shapiro, 1999; Belchamber and Donnelly, 2017). Also, many studies on colonic diseases reported that macrophages release high levels of inflammatory mediators, driving recruitment of other inflammatory cells to the colon and promoting disease progression (Pender et al., 2006; Heinsbroek and Gordon, 2009; Smith et al., 2011). As suggested above, macrophages are considered to play a pivotal role in pulmonary and intestinal inflammatory diseases.

In this study, we again observed pulmonary and intestinal inflammatory response using mice model with cigarette smoke. Investigating the production of inflammatory cytokines, chemokine, and proteases, we examined how macrophage depletion by clodronate liposome treatment influence the inflammation in the lung and colon from CS exposure.

It is well known in the current literature that CS disturbs the lung homeostasis. The mechanism of CS in causing intestinal homeostatic imbalance has also been illustrated (Verschuere et al., 2012). Our recent study has suggested a possible mechanism by which colitis is induced from CS (Lee et al., 2017). In the current study, pulmonary and intestinal inflammation was induced by CS exposure using the 3R4F reference cigarette in C57BL/6 mice as in our previous study. Macrophage depletion state was made by the injection of clodronate containing liposome, and empty liposome treated mice were used as the negative control. Body weight loss is a typical feature of the two representative inflammatory diseases, COPD and colitis (Wack and Rodin, 1982; Muers and Green, 1993; Torres et al., 2017). After 4 weeks of CS exposure, the body weights of mice with empty liposome were significantly decreased. However, the mice with clodronate liposome showed only a slight decrease in body weights. We also confirmed that CS exposure induced the recruitment of inflammatory cells including neutrophils, macrophages, and lymphocytes in BALF. Furthermore, abnormal alterations in the lung from CS exposure, including the migration of immune cells into the perivascular and peribronchial area and the emphysematous changes, were confirmed. In macroscopic and histologic examination in the colon, shortened colon length and mucosal edema by cell infiltration were identified. In addition, we also confirmed CS exposure increased populations of macrophage and dendritic cells (DC) in the colonic lamina propria. While CS-exposure provoked the inflammatory condition of the lung and colon in the empty liposome injected mice, its effect was alleviated with clodronate liposome treatment. We also examined the levels of inflammatory cytokines, chemokine and matrix metalloproteinases in our study, and confirmed that the levels of cytokines and chemokine in BALF and the colonic tissue were elevated. IL-1β is an inflammatory cytokine produced by activated macrophages, and is a central factor in the development of COPD and colonic inflammation. IL-1β stimulates alveolar macrophages activation, and promotes MMPs expression, causing airway destruction and emphysema. Moreover, IL-1β also causes up-regulation of IL-6 and promoting neutrophil recruitment and activation (Okuno et al., 2002; Lappalainen et al., 2005; Churg et al., 2009; Coccia et al., 2012). IL-6 signaling is very important to pulmonary and intestinal inflammation, and is present in high concentrations not only in the sputum of COPD patients but also in the serum of IBD patients. IL-6 is required for mucus hypersecretion and the development of elastase-induced lung tissue remodeling (Tasaka et al., 2010; Rincon and Irvin, 2012). As for colonic inflammation, IL-6 induces macrophage recruitment and activation, and promotes protease expression, causing abnormal changes in the colon (Strober and Fuss, 2011; Bernardo et al., 2012). Besides, TNF-α is an important cytokine, which is reported to be implicated in both COPD and Crohn's disease (CD). TNF-α induces macrophage activation, mucus secretion, and protease secretion, which are responsible for mucosal tissue destruction (Plevy et al., 1997; Mukhopadhyay et al., 2006). MCP-1 prompts the recruitment of macrophages into the airway and colonic tissue, and stimulates protease expression (Antoniades et al., 1992; Banks et al., 2003; Yang et al., 2014).

Produced by macrophages or other immune cells stimulated by macrophage cytokines, matrix metalloproteinases are proteolytic enzymes that contribute to the degradation of mucosal structural components. DC is an antigen presenting cell with phagocytosis. Numbers of DC are increased in the colonic tissue of colitis mice, and DCs are involved in the development of colitis by contributing to productions of Th1 cytokines (Berndt et al., 2007).

It is known that MMPs' overexpression exacerbates tissue damage during mucosal inflammation such as COPD and CD (Bamba et al., 2003; Vernooy et al., 2004; Nenan et al., 2005; Siloşi et al., 2014). The expression of MMP-3 and MMP-9 are significantly increased in the inflamed colonic mucosa of COPD and IBD patients. It has been shown that the lack of MMP-3 and MMP-9 alleviate pathological changes and related injuries in the lung (Korytina et al., 2012). MMP-12, macrophage elastase, is also involved in extracellular matrix degredation and is associated with the development of emphysema and the increase in intestinal tight junction permeability in IBD (Molet et al., 2005; Pender et al., 2006; O'Sullivan et al., 2015).

It was observed in this study that clodronate liposome treatment ameliorates the CS-induced inflammatory conditions, lowers the level of cytokines and chemokine, and reduces the over-expression of MMPs in both the lung and colon, and protects macrophage and DC accumulations in the colonic lamina propria by macrophage depletion.

In summary, our results demonstrated that activated macrophages and resulting inflammatory mediators, including cytokines, chemokine, and proteases, play a critical role in the cigarette smoke-induced pulmonary and intestinal inflammatory response, and that the injection of clodronate containing liposome alleviated this CS-induced inflammatory response in mice through the macrophage depletion and the resulting inflammatory mediators. Therefore, we suggest that clodronate liposome may be a candidate resource for attenuating pulmonary and intestinal inflammation. However, there is still a possibility that macrophage depletion may shift the immune system out of balance. It has been shown that macrophage depletion leads to a different pro-inflammatory cytokine activation from viral pathogens and is associated with infection susceptibility (Murphy et al., 2011). Nevertheless, other studies show contrasting results. Further research is therefore necessary in order to improve the therapeutic use of macrophage depletion.

## Author contributions

DL performed the majority of experiments and wrote the manuscript; WK contributed to the acquisition of data; CL performed the flow cytometry and immunohistochemistry analysis; HB analyzed the flow cytometry and immunohistochemistry data; JK designed the research and analyzed the data.

### Conflict of interest statement

The authors declare that the research was conducted in the absence of any commercial or financial relationships that could be construed as a potential conflict of interest.

## Supplementary material

Supplementary Figure 1 Changes with cell infiltration in bronchoalveolar lavage fluid (BALF). The cells were confirmed with light microscopy after Diff-Quick staining. EL CON(*n* = 6): control mice with empty liposome; EL CS(*n* = 8): CS-exposed mice with empty liposome; CL CON(*n* = 6): control mice with clodronate containing liposome; CL CS(*n* = 12): CS-exposed mice with clodronate containing liposome. Magnification × 400.
