# Supplementary figures and images for: Macrophage Depletion Protects against Cigarette Smoke-Induced Inflammatory Response in the Mouse Colon and Lung
Source: Front Physiol. 2018 Feb 12;9:47. doi: 10.3389/fphys.2018.00047 (PMC5816061; doi:10.3389/fphys.2018.00047)

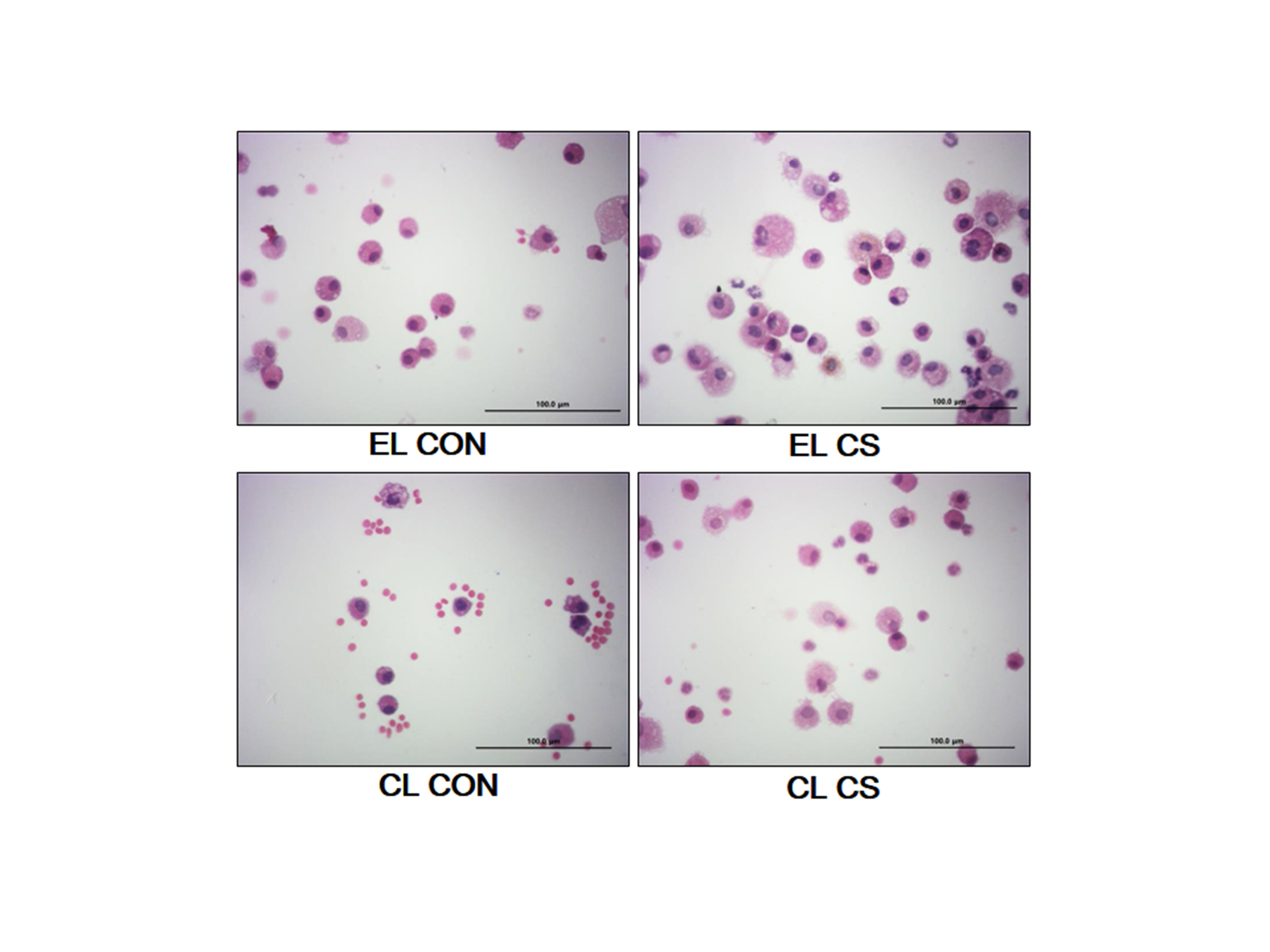

Supplement: Supplementary Figure 1 — Changes with cell infiltration in bronchoalveolar lavage fluid (BALF). The cells were confirmed with light microscopy after Diff-Quick staining. EL CON(n = 6): control mice with empty liposome; EL CS(n = 8): CS-exposed mice with empty liposome; CL CON(n = 6): control mice with clodronate containing liposome; CL CS(n = 12): CS-exposed mice with clodronate containing liposome. Magnification × 400. [file Image1.TIF]
